# Chromosome-level genome assemblies of *Nicotiana tabacum*, *Nicotiana sylvestris*, and *Nicotiana tomentosiformis*

**DOI:** 10.1038/s41597-024-02965-2

**Published:** 2024-01-26

**Authors:** Nicolas Sierro, Mehdi Auberson, Rémi Dulize, Nikolai V. Ivanov

**Affiliations:** PMI R&D, Philip Morris Products S.A., Quai Jeanrenaud 5, CH-2000 Neuchâtel, Switzerland

**Keywords:** Comparative genomics, Polyploidy in plants

## Abstract

The Solanaceae species *Nicotiana tabacum*, an economically important crop plant cultivated worldwide, is an allotetraploid species that appeared about 200,000 years ago as the result of the hybridization of diploid ancestors of *Nicotiana sylvestris* and *Nicotiana tomentosiformis*. The previously published genome assemblies for these three species relied primarily on short-reads, and the obtained pseudochromosomes only partially covered the genomes. In this study, we generated annotated *de novo* chromosome-level genomes of *N. tabacum*, *N. sylvestris*, and *N. tomentosiformis*, which contain 3.99 Gb, 2.32 Gb, and 1.74 Gb, respectively of sequence data, with 97.6%, 99.5%, and 95.9% aligned in chromosomes, and represent 99.2%, 98.3%, and 98.5% of the near-universal single-copy orthologs Solanaceae genes. The completion levels of these chromosome-level genomes for *N. tabacum*, *N. sylvestris*, and *N. tomentosiformis* are comparable to other reference Solanaceae genomes, enabling more efficient synteny-based cross-species research.

## Background & Summary

The *Nicotiana* genus belongs to the Solanaceae family, which also includes tomato (*Solanum lycopersicum*), potato (*Solanum tuberosum*), and eggplant (*Solanum melongena*)^1,2^. While most of the Solanaceae are diploids with 12 chromosome pairs, tobacco (*Nicotiana tabacum* L.) is an allotetraploid (2n = 4x = 48) resulting from a hybridization event that likely occurred in the Andes within the last 200,000 years between ancestors of *Nicotiana sylvestris* (S-genome; 2n = 2x = 24) and *Nicotiana tomentosiformis* (T-genome; 2n = 2x = 24)^3,4^. In addition to being a modern descendant of the *N. tabacum* maternal progenitor, *N. sylvestris*, which is nowadays largely cultivated as an ornamental plant, is also one the closest descendants of the ancestral species from the *Alatae/Sylvestres* section that hybridized as the paternal donor with an ancestral species from the *Noctiflorae/Petunioides* section to give rise to the almost all-Australian clade of allopolyploid species constituting the *Nicotiana* section *Suaveolentes*^5^.

Similar to other members of the *Nicotiana* genus, *N. sylvestris*, *N. tomentosiformis*, and *N. tabacum* produce a wide range of alkaloids that are known to be toxic to insects and are a well-established mechanism of defense against herbivores^6^. While *N. sylvestris* accumulates similar amounts of alkaloids in roots and leaves (3.5 mg/g in roots and 2.1 mg/g in leaves), *N. tomentosiformis* accumulates more alkaloids in roots (8.8 mg/g in roots and 0.6 mg/g in leaves), and *N. tabacum* has more in leaves (1.3 mg/g in roots and 12.5 mg/g in leaves)^7^. The composition of the accumulated alkaloids varies between the three species, with *N. tabacum* benefiting from both of its progenitors’ genetic and regulatory contributions. In *N. sylvestris* roots, 87% of the alkaloids is nicotine, 11% is anatabine, and 1.9% is anabasine, while in leaves, 100% of the alkaloids is nicotine. In *N. tomentosiformis* roots, 56% of the alkaloids is nornicotine, 28% is anatabine, 14% is nicotine, 1.6% is anabasine, and 0.57% is cotinine, while in leave 73% of the alkaloids is nicotine and 27% is nornicotine. In *N. tabacum* roots, 87% of the alkaloids is nicotine, and 13% is nornicotine, while in leaves, 92% of the alkaloids is nicotine, 5.1% is nornicotine, and 2.6% is anatabine^7^.

The *Nicotiana* genus is also a rich source of terpenoids, which play a significant role as attractants to several pollinator insects. In *N. tabacum*, both cembranoid and labdanoid diterpenoids are synthesized in the trichome glands, whereas *N. sylvestris* produces predominantly cembranoid diterpenoids and *N. tomentosiformis* predominantly labdanoid diterpenoids^8^.

Although several *Nicotiana* species genomes have been published in the last decade, including for *N. sylvestris*^9^, *N. tomentosiformis*^9^, and *N. tabacum*^10,11^, these genomes are primarily based on the assembly of second-generation sequencing data and therefore suffer from an important fragmentation resulting in only partial anchoring to chromosomes.

In the present study, we integrated Illumina short-read sequencing (Illumina, San Diego, CA, USA) with third-generation Oxford Nanopore long-read sequencing and Oxford Nanopore chromosome conformation capture (PoreC) technology (Oxford Nanopore Technologies, Oxford, UK) to generate high-quality chromosome-level reference genomes for *N. tabacum*, *N. sylvestris*, and *N. tomentosiformis*. These new resources will broaden our understanding of the contributions of both *N. tabacum* progenitors to the genes and the pathways of tobacco and enable more efficient synteny-based cross-species Solanaceae research.

## Methods

### DNA Extraction and Sequencing

Young leaves from *N. tabacum* L. Cultivar K326 (PVY resistant derived from USDA ARS GRIN Global NPGS: PI 552505), *N. Sylvestris* Speg. TW136 (USDA ARS GRIN Global NPGS: PI 555569) and *N. tomentosiformis* Goodsp. TW142 (USDA ARS GRIN Global NPGS: PI 555572) were snap-frozen with liquid nitrogen and finely ground in a mortar. High molecular weight genomic DNA for long-read sequencing was extracted using Promega Wizard HMW DNA Extraction Kit (Promega AG, Madison, WI, USA).

Short genomic DNA fragments were deleted using Circulomics short-read eliminator kits from PacBio (PacBio, Menlo Park, CA, USA), and long-read sequencing libraries were prepared using Oxford Nanopore Technologies SQK-LSK109 Ligation Sequencing Kits before sequencing on Oxford Nanopore Technologies PromethION R9.4.1 flowcells. About 139 Gb of raw data were collected for *N. tabacum*, 159 Gb for *N. sylvestris*, and 76 Gb for *N. tomentosiformis*.

To conduct chromosome-level assembly, frozen leaves were cut into one square centimeter pieces and treated with formaldehyde to fix the DNA. The fixed genomic DNA was then digested overnight using the NlaIII restriction enzyme, and the 3′ overhangs were re-ligated using T4 ligase before extraction. PoreC sequencing libraries were prepared using Oxford Nanopore Technologies SQK-LSK109 Ligation Sequencing Kits before sequencing on Oxford Nanopore Technologies PromethION R9.4.1 flowcells. About 40 Gb of raw data were collected for *N. tabacum*, 66 Gb for *N. sylvestris*, and 63 Gb for *N. tomentosiformis*.

To polish and validate the assembled genomes, Illumina short-reads were prepared for *N. tabacum* using Tecan Celero EZ DNA-Seq Library Preparation Kits (Tecan, Männedorf, Switzerland) and sequenced as 2 × 151 bp paired-end reads on an Illumina NovaSeq 6000 to generate a total of 139 Gb. Illumina short-reads from ERR274527^12^ and ERR274528^13^ for *N. sylvestris* and from ERR274540^14^ and ERR274542^15^ for *N. tomentosiformis* were retrieved from the Short Read Archive.

### *De novo* Assembly and Chromosome Construction

For *N. tabacum*, Oxford Nanopore basecalling was performed using Guppy 6.3.7 using the plant super model. Long-read sequences were filtered using seqkit^16^ 2.2.0 to remove short (length <5000) and low-quality reads (average qscore <9), resulting in 98 Gb (N50 length: 28.5 kb).

For *N. sylvestris* and *N. tomentosiformis*, Oxford Nanopore basecalling was performed using Guppy 6.1.1 using the plant super model. Long-read sequences were filtered using seqkit^16^ 2.2.0 to remove short (length <2500) and low-quality reads (average qscore <9), resulting in 108 Gb (N50 length: 25.9 kb) and 41 Gb (N50 length: 28.2 kb) for *N. sylvestris* and *N. tomentosiformis*, respectively.

Genomes were assembled using flye^17^ 2.9.1 using the nano-hq input pre-set and a read error rate of 0.03.

The Illumina short-reads were processed for each species using fastp^18^ 0.23.2 to trim adapters and low-quality bases, merge pairs, and remove low complexity and short (length <75) reads. During processing, the reads were split into two sets, one for assembly polishing which contained 80% of the processed Illumina reads and one for assembly validation containing 20% of the processed Illumina reads.

The assembled genomes were polished with processed Illumina short-reads using fmlrc2^19^ 0.1.7. The remaining haplotig sequences were removed from the assemblies using purge_dups^20^ 1.2.6, with cut-offs set to 3, 8, and 1000 for *N. tabacum*, to 5, 10, and 1000 for *N. sylvestris*, and to 2, 3, and 1000 for *N. tomentosiformis*.

Illumina short-reads were mapped to the assembly contigs using minimap2^21,22^ 2.24, duplicates marked with samblaster^23^ 0.1.26, and filtered using samtools^24^ 1.15.1. The coverage of the assembly contigs by Illumina sequencing was then calculated using samtools^24^ 1.15.1, and contigs with less than 70% of their length with a coverage of at least 5 for *N. tabacum* and 15 for *N. sylvestris* and *N. tomentosiformis* were removed.

Because the biological material used for sequencing originated from inbred plants that can be considered homozygotes, variants were called using freebayes^25^ 1.3.6 with the ploidy parameter set to 1 and ignoring sites with coverage higher than 200 and filtered with vcflib^26^ 1.0.3 vcffilter using the parameters --filter-sites–info --filter “QUAL >20 & QUAL/AO >10 & SAF >0 & SAR >0 & RPL >1 & RPR >1”. Variants were then applied to the genomes using bcftools^24^ 1.15.1 consensus to generate the polished assembly contigs.

Assembly contigs from plastid and mitochondrion were removed by mapping the polished assembly contigs to the *N. tabacum* plastid and mitochondrion sequences (NC_001879.2^27^ and NC_006581.1^28^, respectively) using minimap2^21,22^ 2.24 and filtering out contig mapping on more than 50% of their length.

Assembly contigs from possible contamination were identified using kraken2^29^ 2.1.2 using the k2_pluspfp_20220908 database^30^ and removed by only retaining contigs identified as belonging to *Nicotiana* or *Solanum* species.

PoreC reads were mapped to the cleaned assembly contigs using minimap2^21,22^ 2.24. Alignments with a mapping quality lower than 60 for *N. tabacum* and 30 for *N. sylvestris* and *N. tomentosiformis* were discarded, and contact pairs were created from the remaining alignments. The positions on the contigs of each contact pair were recorded as two consecutive lines in a BED file. The scaffolding of the contigs to a chromosome-level assembly was performed using yahs^31^ 1.2a1. Contact maps were prepared using PretextMap^32^ 0.1.9, manually curated and annotated in PretextView^33^ 0.2.5, and the resulting scaffolds exported as chromosome-level sequences.

**Fig. 1:**
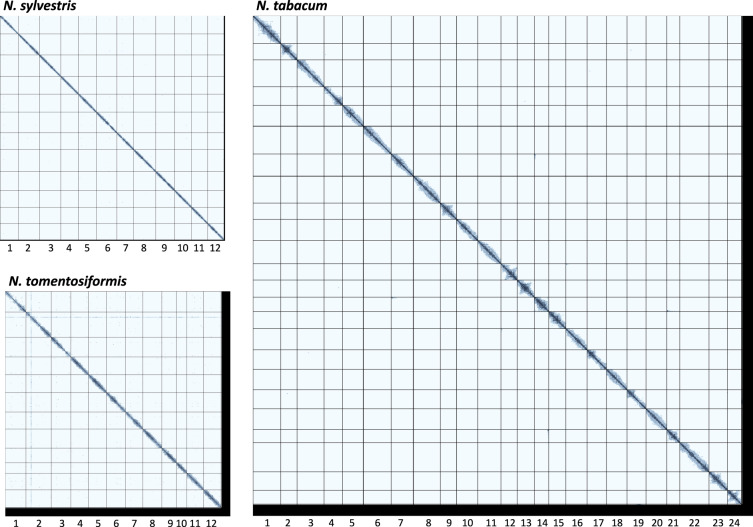
PoreC contact maps. Intra-chromosomal and inter-chromosomal contacts are shown for the *Nicotiana sylvestris*, *Nicotiana tomentosiformis*, and *Nicotiana tabacum* genome assemblies. The black bottom and right edges correspond to unplaced sequences.

To name and orient the *N. tabacum* chromosome-level sequences, the PT markers, mapped to the sequences using hisat2^34^ 2.2.1 and the tobacco genetic map^35^, were used. Similarly, the *N. tomentosiformis* chromosome-level sequences were named and oriented using the N genetic map^36^ combined with the tobacco PT markers^35^. The chromosome-level assembly of the *N. tomentosiformis* genome was then used as a reference to name and orient the *N. sylvestris* chromosome-level sequences based on minimap2^21,22^ 2.24 mapping (Fig. 1).

The proportion of the assembly anchored to chromosomes reached 99.5%, 95.9%, and 97.6% of the total assembly lengths for *N. sylvestris*, *N. tomentosiformis*, and *N. tabacum*, respectively (Table 1).

**Table 1:** Chromosome length, total assembly length, and percentage of the assembly anchored to chromosomes for *Nicotiana sylvestris, Nicotiana tomentosiformis*, and *Nicotiana tabacum*.

	*N. sylvestris*	*N. tomentosiformis*	*N. tabacum*
Chr01	188,594,255	159,904,673	222,086,288
Chr02	216,772,750	195,009,794	130,336,781
Chr03	222,355,857	151,524,687	215,112,738
Chr04	182,174,535	137,929,616	150,061,924
Chr05	183,858,385	139,001,852	166,564,982
Chr06	213,680,027	150,012,402	218,894,441
Chr07	174,301,312	131,654,490	180,540,203
Chr08	226,073,828	146,876,285	212,334,375
Chr09	193,471,688	113,607,955	128,373,858
Chr10	173,459,395	80,117,918	173,455,358
Chr11	166,895,819	128,153,117	182,075,898
Chr12	168,333,862	136,938,251	131,632,239
Chr13			135,455,135
Chr14			117,149,023
Chr15			135,283,689
Chr16			171,654,204
Chr17			153,689,555
Chr18			161,231,186
Chr19			151,880,626
Chr20			168,699,142
Chr21			101,618,745
Chr22			234,076,610
Chr23			146,657,309
Chr24			112,906,552
Unplaced	11,613,273	71,022,666	93,985,334
Total	2,321,584,986	1,741,753,706	3,995,756,195
% anchored	99.5%	95.9%	97.6%

When compared to the previously available *N. tabacum* genome assembly^11^ generated from short-read sequencing, whole genome profiling and optical and genetic mapping data, the new *N. tabacum* genome assembly has fewer contigs (decrease from 1,257,801 to 1410) with a larger N50 length (increase from 9.1 kb to 11.8 Mb), and the proportion of the assembly anchored to chromosomes consequently improved from 64% to 97.6%.

### Retrotransposon Prediction and Annotation

Nested retrotransposons were annotated by iteratively running genometools 1.6.2 ltrharvest^37^ using the parameters -similar 70 -seed 20 -minlenltr 100 -maxlenltr 7000 -mindistltr 1000 -maxdistltr 15000 -mintsd 4 -maxtsd 6 -motif TGCA -motifmis 3 -vic 10 -overlaps best, retaining the predictions matching to the RepeatExplorer Viridiplantae 3.0 dataset^38^ using diamond^39^ 2.1.6 blastx with the parameters --max-target-seqs 1 --ultra-sensitive --frameshift 15, and excising them from the assembly using samtools^24^ 1.17. At most, 20 prediction-filtering-excision iterations were performed.

The predicted retrotransposons were classified by their homology to the RepeatExplorer Viridiplantae 3.0 dataset^38^ sequences. Their age was estimated under the assumption that their long terminal repeats (LTRs) were identical at the time of insertion by aligning their 3′ and 5′ LRTs using clustalo^40,41^ 1.2.4, calculating their divergence (K) using the Kimura-2-parameter distance and dividing it by twice 1.5 × 10^−8^ substitution per site per year (r)^42^.

**Fig. 2:**
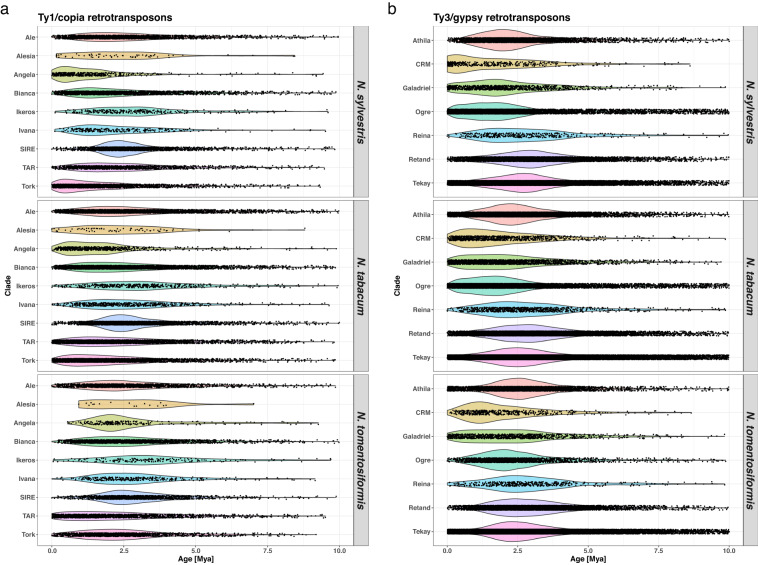
Predicted retrotransposon insertion ages. (**a**) Predicted insertion ages in millions of years for retrotransposons of the Ty1/copia superfamily; (**b**) Predicted insertion ages in millions of years for retrotransposons of the Ty3/gypsy superfamily.

The predicted retrotransposons covered 26.6%, 32.2%, and 29.3% of the *N. sylvestri*s, *N. tomentosiformis*, and *N. tabacum* genomes, respectively (Table 2). Regardless of the species, the most frequent element subclass is Ty3/gypsy|chromovirus|Tekay, representing between 40% and 56% of the total predicted retrotransposon length. The only element subclass that shows a marked difference between the three species is Ty3/gypsy|non-chromovirus|OTA|Tat|Ogre, which covers 116,167,517 bp (18.8% of the total predicted retrotransposon length) in *N. sylvestris*, and only 21,672,795 bp (3.9%) in *N. tomentosiformis*. In *N. tabacum*, it covers 135,653,424 bp (11.6%), close to the sum of its coverage in the two precursor species (137,840,312 bp). Looking at the predicted insertion ages, a recent expansion of the Alesia and Angela subclasses of Ty1/copia and of the Ogre subclass of Ty3/gypsy retrotransposons in *N. sylvestris* and *N. tabacum*, but not in *N. tomentosiformis*, is observed (Fig. 2).

**Table 2:** Predicted retrotransposons length and genome coverage statistics.

				*N. sylvestris*			*N. tomentosiformis*			*N. tabacum*		
				length	% of TE	% of genome	length	% of TE	% of genome	length	% of TE	% of genome
Class I	LINE			9,675,761	1.6%	0.4%	9,615,294	1.7%	0.6%	19,745,491	1.7%	0.5%
	LTR	Ty1/copia	Ale	13,558,929	2.2%	0.6%	11,670,567	2.1%	0.7%	24,896,186	2.1%	0.6%
	LTR	Ty1/copia	Alesia	299,556	0.0%	0.0%	102,813	0.0%	0.0%	380,375	0.0%	0.0%
	LTR	Ty1/copia	Angela	3,989,230	0.6%	0.2%	1,986,947	0.4%	0.1%	5,826,451	0.5%	0.1%
	LTR	Ty1/copia	Bianca	14,202,928	2.3%	0.6%	12,796,742	2.3%	0.7%	26,200,095	2.2%	0.7%
	LTR	Ty1/copia	Ikeros	2,115,094	0.3%	0.1%	1,471,616	0.3%	0.1%	3,637,043	0.3%	0.1%
	LTR	Ty1/copia	Ivana	1,366,613	0.2%	0.1%	1,775,454	0.3%	0.1%	3,081,268	0.3%	0.1%
	LTR	Ty1/copia	SIRE	24,828,773	4.0%	1.1%	14,684,980	2.6%	0.8%	38,903,674	3.3%	1.0%
	LTR	Ty1/copia	TAR	9,154,238	1.5%	0.4%	12,202,057	2.2%	0.7%	20,574,287	1.8%	0.5%
	LTR	Ty1/copia	Tork	14,900,248	2.4%	0.6%	7,980,408	1.4%	0.5%	21,895,949	1.9%	0.5%
	LTR	Ty3/gypsy	chromovirus|CRM	2,617,599	0.4%	0.1%	2,797,010	0.5%	0.2%	5,522,890	0.5%	0.1%
	LTR	Ty3/gypsy	chromovirus|Chlamyvir	0	0.0%	0.0%	0	0.0%	0.0%	5,786	0.0%	0.0%
	LTR	Ty3/gypsy	chromovirus|Galadriel	5,700,997	0.9%	0.2%	5,500,007	1.0%	0.3%	11,145,772	1.0%	0.3%
	LTR	Ty3/gypsy	chromovirus|Reina	2,340,197	0.4%	0.1%	2,730,019	0.5%	0.2%	4,966,891	0.4%	0.1%
	LTR	Ty3/gypsy	chromovirus|Tcn1	0	0.0%	0.0%	0	0.0%	0.0%	3,227	0.0%	0.0%
	LTR	Ty3/gypsy	chromovirus|Tekay	248,756,499	40.3%	10.7%	310,558,487	55.4%	17.8%	559,106,371	47.7%	14.0%
	LTR	Ty3/gypsy	chromovirus|chromo-outgroup	0	0.0%	0.0%	5,356	0.0%	0.0%	15,594	0.0%	0.0%
	LTR	Ty3/gypsy	non-chromovirus|OTA|Athila	59,196,881	9.6%	2.5%	50,111,064	8.9%	2.9%	108,359,430	9.3%	2.7%
	LTR	Ty3/gypsy	non-chromovirus|OTA|Tat|Ogre	116,167,517	18.8%	5.0%	21,672,795	3.9%	1.2%	135,653,424	11.6%	3.4%
	LTR	Ty3/gypsy	non-chromovirus|OTA|Tat|Retand	75,130,400	12.2%	3.2%	81,189,167	14.5%	4.7%	155,002,488	13.2%	3.9%
	pararetrovirus			6,158,720	1.0%	0.3%	3,683,969	0.7%	0.2%	9,724,069	0.8%	0.2%
Class II	Subclass 1	TIR	EnSpm/CACTA	758,007	0.1%	0.0%	1,412,566	0.3%	0.1%	2,030,473	0.2%	0.1%
	Subclass 1	TIR	MuDR/Mutator	2,475,225	0.4%	0.1%	1,382,863	0.2%	0.1%	4,014,994	0.3%	0.1%
	Subclass 1	TIR	PIF/Harbinger	114,448	0.0%	0.0%	128,968	0.0%	0.0%	294,983	0.0%	0.0%
	Subclass 1	TIR	Tc1/Mariner	26,044	0.0%	0.0%	100,923	0.0%	0.0%	83,809	0.0%	0.0%
	Subclass 1	TIR	hAT	3,476,633	0.6%	0.1%	3,931,259	0.7%	0.2%	7,522,070	0.6%	0.2%
	Subclass 2	Helitron	Helitron	860,377	0.1%	0.0%	1,440,401	0.3%	0.1%	23,79,749	0.2%	0.1%
Total				617,870,914	100.0%	26.6%	560,931,732	100.0%	32.2%	1,170,972,839	100.0%	29.3%

### Coding-gene Prediction and Annotation

Genomes were masked using blast^43,44^ 2.14.0 windowmasker with dusting, and augustus^45^ 3.5.0 was used for gene prediction. A training dataset was created by separately mapping *S. lycopersicum*, *S. tuberosum*, and *Nicotiana attenuata* cDNA and CDS from Ensembl 56 using minimap2^21,22^ 2.26 to the *N. sylvestris* and *N. tomentosiformis* genomes. Any sequence with an annotation matching ‘hypothetical’, ‘unknown’, ‘polyprotein’, ‘domain-containing’, ‘chloroplast’, or ‘mitochondria’ were omitted from the mapping. Gene models were constructed from the mapped sequences using bedtools^46^ 2.30.0 and filtered using gffread^47^ 0.12.7 with the parameters -V -H -U -N -P -J -M -K -Q -Y -Z -F --keep-exon-attrs. Training sequences were then extracted from the genomes using the obtained GFF annotation file and adding 1,000 bp flaking regions. One-fourth of the gene models were set aside for testing for each combination of species and dataset. After merging the training and testing datasets, a Nicotiana model was trained using the etraining and optimize_augustus.pl programs bundled with augustus^45^ 3.5.0. A total of 10,092 loci were used for training, and 3,362 loci were used for testing.

To hint at the augustus predictions, Ensembl 56 proteins from *S. lycopersicum*, *S. tuberosum*, and *N. attenuata* were mapped to the genomes using miniprot^48^ 0.11, and aletsch^49^ 1.0.3 was used to construct transcripts from Illumina paired-end RNA-Seq reads from SRR11912457^50^, SRR2106531^51^, ERR274387^52^, ERR274388^53^, ERR274389^54^, ERR274390^55^, ERR274391^56^, ERR274392^57^, ERR274393^58^, ERR274394^59^, ERR274395^60^, ERR274396^61^, ERR274397^62^, ERR274398^63^, ERR274399^64^, ERR274400^65^, ERR274401^66^, ERR274402^67^, ERR274403^68^, ERR274404^69^, and ERR274405^70^ mapped using hisat2^34^ 2.2.1, and Oxford Nanopore long cDNA reads from SRR12045991^71^, SRR12045992^72^, SRR12045993^73^, and SRR12045994^74^ mapped with minimap2^21,22^ 2.26.

Augustus^45^ 3.5.0 predictions were obtained using the trained Nicotiana model, the extrinsic.MPE.cfg extrinsic configuration file, and hints derived from the miniport^48^ 0.11 and aletsch^49^ 1.0.3 output with priorities of 4 and 3, respectively. Other augustus^45^ 3.5.0 parameters used were --alternatives-from-evidence=off --alternatives-from-sampli ng=off --softmasking=1 --strand=both --genemodel=complete --UTR=on. Predicted gene models without supporting hints that did not encode a protein found in a uniprot eudicotyledons proteins dataset filtered to omit proteins with annotations matching ‘uncharacterized’, ‘unknown’, ‘hypothetical’, ‘genome’, ‘domain-containing’, ‘family’, ‘transmembrane’, ‘putative’, ‘probable’, ‘predicted’, ‘member’, ‘fragment’, ‘truncated’, ‘superfamily’, ‘chloroplast’, ‘mitochond’, ‘low quality’, or ‘At.g’ when using diamond^39^ 2.1.6 blastx with the parameters --max-target-seqs 1 --min-score 200 --ultra-sensitive --frameshift 15 were removed.

To complement the augustus predictions, additional gene models were created by separately mapping the predicted *N. sylvestris*, *N. tomentosiformis*, and *N. tabacum* cDNA and CDS and the *S. lycopersicum*, *S. tuberosum*, and *N. attenuata* cDNA and CDS from Ensembl 56 to the genomes using minimap2^21,22^ 2.26. Models that overlapped augustus predictions by 25% or more according to bedtools^46^ 2.30.0 intersect were then filtered out by IDs using gffread^47^ 0.12.7 with the parameters -P -M -K -Q -Y -Z -F, and the remaining genes models were added to those predicted with augustus^45^ 3.5.0.

Functional annotation of the gene models was performed using diamond^39^ 2.1.6 blastx with the parameters --max-target-seqs 1 --min-score 200 --ultra-sensitive --frameshift 15 and uniprot eudicotyledons proteins filtered to omit proteins with annotations matching ‘uncharacterized’, ‘unknown’, ‘hypothetical’, ‘genome’, ‘domain-containing’, ‘family’, ‘transmembrane’, ‘putative’, ‘probable’, ‘predicted’, ‘member’, ‘fragment’, ‘truncated’, ‘superfamily’, ‘chloroplast’, ‘mitochond’, ‘low quality’ or ‘At.g’. Gene models overlapping with retrotransposons by 75% or more according to bedtools^46^ 2.30.0 intersect and those with annotations matching ‘transposon’, ‘transposase’, ‘polyprotein’, ‘gagpol’, or ‘gag-pol’ were excluded to yield the final set of annotated gene models.

## Data Records

The genomes and annotations are available from Zenodo under records 8256252^75^, 8256254^76^, and 8256256^77^. The trained Nicotiana model for augustus gene prediction is available from Zenodo under record 8256280^78^.

The genomes have been deposited at DDBJ/ENA/GenBank under the accessions ASAF00000000^79^, ASAG00000000^80^ and AWOJ00000000^81^.

Raw sequencing data are available from the National Center for Biotechnology Information Short Read Archive under accessions SRR25685126^82^, SRR25685127^83^, SRR25685128^84^, SRR25685129^85^, and SRR25685130^86^ in BioProject PRJNA182500, SRR25685034^87^, SRR25685035^88^, SRR25685036^89^, SRR25685037^90^, SRR25685038^91^, SRR25685039^92^, and SRR25685040^93^ in BioProject PRJNA182501, and SRR25685386^94^, SRR25685387^95^, SRR25685388^96^ SRR25685389^97^, SRR25685390^98^, SRR25685391^99^, SRR25685392^100^, SRR25685393^101^, SRR25685394^102^, SRR25685395^103^, and SRR25685396^104^ in BioProject PRJNA208210 for *N. sylvestris*, *N. tomentosiformis*, and *N. tabacum*, respectively.

## Technical Validation

The quality and completeness of the assemblies were assessed with yak^105^ 0.1 using 20% of the processed Illumina short-reads which were set aside for that purpose. For *N. tabacum*, Quality Coverage and Quality Value of 0.982 and 38.1 were obtained; for *N. sylvestris*, they were of 0.993 and 41.5; and for *N. tomentosiformis* they were of 0.991 and 43.2.

The quality of the gene predictions from the trained Nicotiana model was evaluated using the prepared testing sets and compared with results obtained using already available models for arabidopsis, tomato, and coyote_tobacco models (Table 3).

**Table 3:** Augustus testing metrics with the arabidopsis, tomato, coyote_tobacco, and Nicotiana models.

Metric	arabidopsis	tomato	coyote_tobacco	Nicotiana
Without hints				
Base_level_sensitivity	0.964	0.971	0.959	**0.976**
Base_level_specificity	0.887	0.917	**0.93**	0.929
Exon_level_sensitivity	0.841	0.857	0.822	**0.872**
Exon_level_specificity	0.731	0.802	0.812	**0.832**
Gene_level_sensitivity	0.335	0.408	0.371	**0.443**
Gene_level_specificity	0.29	0.369	0.367	**0.418**
UTR_nucleotide_level_sensitivity	**0.623**	0.475	0.434	0.475
UTR_nucleotide_level_specificity	0.455	0.487	0.492	**0.557**
UTR_exon_level_sensitivity	**0.183**	0.16	0.151	0.17
UTR_exon_level_specificity	0.162	0.159	0.177	**0.185**
Accuracy	0.745533	0.7844	0.771333	**0.804933**
With hints				
Base_level_sensitivity	0.987	0.991	0.991	**0.992**
Base_level_specificity	0.945	0.953	**0.965**	0.959
Exon_level_sensitivity	0.955	0.954	0.953	**0.956**
Exon_level_specificity	0.904	0.915	**0.926**	0.924
Gene_level_sensitivity	**0.751**	0.742	0.737	0.749
Gene_level_specificity	0.695	0.696	**0.719**	0.718
UTR_nucleotide_level_sensitivity	**0.598**	0.457	0.417	0.437
UTR_nucleotide_level_specificity	0.612	0.611	**0.706**	0.696
UTR_exon_level_sensitivity	**0.236**	0.21	0.216	0.227
UTR_exon_level_specificity	0.223	0.204	0.236	**0.238**
Accuracy	0.905333	0.908	0.9124	**0.913733**

The best scores are highlighted in bold. Accuracy is calculated as ( 3 × nsen + 2 × nspe + 4 × esen + 3 × espe + 2 × gsen + 1 × gspe )/15.

The completeness of the gene model sets was evaluated using BUSCO^106^ 5.4.7 with the solanales_odb10 lineage dataset. Completeness of 98.1%, 95.1%, and 96.1% at the transcript level and of 97.0%, 92.8%, and 93.4% at the protein level were obtained for *N. tabacum*, *N. sylvestris*, and *N. tomentosiformis*, respectively (Table 4). These values are similar to those obtained for *S. lycopersicum*, of 95.0% at the transcript level and 92.3% at the protein level.

**Table 4:** Statistics of the BUSCO genome, transcripts, and proteins completeness evaluation using the solanales_odb10 lineage dataset for *Nicotiana sylvestris*, *Nicotiana tomentosiformis* and *Nicotiana tabacum*.

	counts			percent		
	Genome	Transcripts	Proteins	Genome	Transcripts	Proteins
*N. sylvestris*						
Complete BUSCOs (C)	5847	5657	5519	98.3%	95.1%	92.8%
Complete and single-copy BUSCOs (S)	5605	5434	5298	94.2%	91.3%	89.0%
Complete and duplicated BUSCOs (D)	242	223	221	4.1%	3.7%	3.7%
Fragmented BUSCOs (F)	8	97	144	0.1%	1.6%	2.4%
Missing BUSCOs (M)	95	196	287	1.6%	3.3%	4.8%
Total BUSCO groups searched	5950	5950	5950	100.0%	100.0%	100.0%
*N. tomentosiformis*						
Complete BUSCOs (C)	5858	5716	5560	98.5%	96.1%	93.4%
Complete and single-copy BUSCOs (S)	5660	5517	5351	95.1%	92.7%	89.9%
Complete and duplicated BUSCOs (D)	198	199	209	3.3%	3.3%	3.5%
Fragmented BUSCOs (F)	12	79	140	0.2%	1.3%	2.4%
Missing BUSCOs (M)	80	155	250	1.3%	2.6%	4.2%
Total BUSCO groups searched	5950	5950	5950	100.0%	100.0%	100.0%
*N. tabacum*						
Complete BUSCOs (C)	5901	5837	5774	99.2%	98.1%	97.0%
Complete and single-copy BUSCOs (S)	525	835	996	8.8%	14.0%	16.7%
Complete and duplicated BUSCOs (D)	5376	5002	4778	90.4%	84.1%	80.3%
Fragmented BUSCOs (F)	1	38	66	0.0%	0.6%	1.1%
Missing BUSCOs (M)	48	75	110	0.8%	1.3%	1.8%
Total BUSCO groups searched	5950	5950	5950	100.0%	100.0%	100.0%

## Data Availability

All software used in this work is publicly available, with versions and parameters clearly described in Methods. If no detailed parameters were mentioned for a software, the default parameters suggested by the developer were used. No custom code was used during this study for the curation and/or validation of the datasets.
